# The effect of loss incentives on prospective memory in healthy older adults: study protocol of a randomized controlled trial using ultra-high field fMRI

**DOI:** 10.1186/s12888-023-05229-2

**Published:** 2023-10-06

**Authors:** Marta Menéndez-Granda, Nadine Schmidt, Michael Orth, Katharina Klink, Sebastian Horn, Matthias Kliegel, Jessica Peter

**Affiliations:** 1 University Hospital of Old Age Psychiatry and Psychotherapy, University of Bern, Bern, Switzerland; 2 Graduate School for Health Sciences, University of Bern, Bern, Switzerland; 3 Swiss Institute for Translational and Entrepreneurial Medicine, Bern, Switzerland; 4 Department of Neurology, Inselspital, Bern University Hospital, University of Bern, Bern, Switzerland; 5 Department of Psychology, University of Zurich, Zurich, Switzerland; 6 Faculty of Psychology and Educational Sciences, University of Geneva, Geneva, Switzerland; 7 Centre for the Interdisciplinary Study of Gerontology and Vulnerability, University of Geneva, Geneva, Switzerland; 8 Swiss Centre of Expertise in Life Course Research, LIVES Centre, Lausanne and Geneva, Switzerland

**Keywords:** Prospective memory, Event-based, Time-based, Healthy ageing, Incentives, Avoidance of losses, Functional MRI

## Abstract

**Background:**

Prospective memory is important for our health and independence but declines with age. Hence, interventions to enhance prospective memory, for example by providing an incentive, may promote healthy ageing. The neuroanatomical correlates of prospective memory and the processing of incentive-related prospective memory changes in older adults are not fully understood. In an fMRI study, we will therefore test whether incentives improve prospective memory in older adults and how prospective memory is processed in the brain in general, and when incentives are provided. Since goals and interests change across adulthood, avoiding losses is becoming more important for older adults than achieving gains. We therefore posit that loss-related incentives will enhance prospective memory, which will be subserved by increased prefrontal and midbrain activity.

**Methods:**

We will include *n* = 60 healthy older adults (60–75 years of age) in a randomized, single-blind, and parallel-group study. We will acquire 7T fMRI data in an incentive group and a control group (*n* = 30 each, stratified by education, age, and sex). Before and after fMRI, all participants will complete questionnaires and cognitive tests to assess possible confounders (e.g., income, personality traits, sensitivity to reward or punishment).

**Discussion:**

The results of this study will clarify whether loss-related incentives can enhance prospective memory and how any enhancement is processed in the brain. In addition, we will determine how prospective memory is processed in the brain in general. The results of our study will be an important step towards a better understanding of how prospective memory changes when we get older and for developing interventions to counteract cognitive decline.

## Background

Prospective memory is the ability to remember doing something at a specific point in time in the future (i.e., time-based prospective memory) or when a specific event occurs (i.e., event-based prospective memory) [1]. Whether brain regions involved in time-based and event-based prospective memory overlap or differ is hardly understood, particularly when it comes to ageing. Most neuroimaging studies in older adults tested event-based prospective memory using fMRI but not time-based prospective memory [2–6]. One study that did assess both types of prospective memory reported that network activity was similar as both tasks activated frontal and parietal brain regions, the insula, and the thalamus [4]. The time-based task in that study, however, was very similar to an event-based task since a clock was always in plain view so the participants did not need to remember the time-based intention by themselves. Instead, they were reminded by an external cue (i.e., the clock) which was constantly visible. Therefore, it could be that brain activity was similar in both tasks just because the task setup was very similar. It would be important to test whether older adults activate other brain regions when they need to remember time-based intentions by themselves [1, 7, 8]. This would help determine whether ageing affects time-based and event-based prospective memory differently in the brain.

Remembering prospective intentions is important for maintaining health and independence in older adults (e.g., remembering to meet a doctor or to take medication at a specific time) [9, 10]. Finding ways to improve or facilitate prospective memory may therefore promote healthy ageing. Motivation influences how well people encode and retrieve memories [11]. Enhancing motivation, for example by providing incentives, may thus be a way to improve prospective memory. Whether and how incentives influence prospective memory in older adults is not fully understood. There is evidence to suggest that event-based prospective memory can be improved when incentives include a prosocial component (i.e., a donation) [12]. This might be particularly motivating for older adults since they are usually more empathic and thus, more prosocial [13]. In addition, there is evidence to suggest that goals and interests change across adulthood [14], with avoiding losses becoming more important for older adults than achieving gains [15]. This is supported by an event-based prospective memory study, in which older adults performed better when they tried to avoid financial losses rather than to achieve financial gains [12]. It would be important to complement insights gained from event-based prospective memory with what happens to time-based prospective memory when losing an incentive was to be avoided. Prospective memory failures in daily life are often followed by financial losses (e.g., forgetting to pay a bill on time or forgetting to return a rented item on time may lead to financial extra charges). A better understanding of the consequences that follow prospective memory failure would therefore help to discern the mechanisms involved in motivational processes of prospective memory. In addition, we know nothing about incentive-related processing in the brain when prospective memory tasks are used. In other cognitive domains, such as decision making, the monetary incentive delay task has often been used to investigate incentive-related processing in the brain [16–18]. It has been found that younger adults activate a fronto-striatal-thalamic network during anticipation of an incentive, regardless of whether they try to achieve gains or to avoid losses [19]. Older adults, in contrast, activate the lateral prefrontal cortex in addition to midbrain areas and the insula [18]. It would be important to test whether these differences in older adults’ activity patterns are specific to the monetary incentive delay task or whether they can be generalised to prospective memory tasks.

The aims of the current study are, therefore, threefold. First, we investigate the functional neuroanatomy of prospective memory with a particular emphasis on the question whether brain regions involved in time-based and event-based prospective memory overlap or differ. We expect them to differ when using a time-based prospective memory paradigm in which participants actively align their intentions with time. Second, we test whether event-based or time-based prospective memory can be modulated when participants have to avoid losing an incentive. We hypothesize that this will improve prospective memory. Third, we investigate the neuroanatomical correlates of incentive-related processing during prospective memory tasks. We expect increased prefrontal and midbrain activity when participants avoid losing an incentive and that the strength of activity increase will be associated with prospective memory task performance.

## Methods and design

### Participants eligibility and recruitment

In this randomized, single-blind, and parallel-group study, *n* = 60 healthy older participants (60—75 years of age) will be included. Inclusion criteria will be fluency in German, no evidence of cognitive impairment on the Cognitive Telephone Screening (COGTEL; [20]), normal or corrected-to-normal vision and no clinically relevant depressive symptoms according to the Geriatric Depression Scale (GDS) (i.e., total score ≤ 5) [21]. Exclusion criteria will be past head injuries, permanent make-up, metal implants above the hips, any history of neurological or psychiatric disease, current or lifetime alcohol or drug abuse, intake of psychotropic drugs, brain damage, as well as magnetisable implants (e.g., cardiac pacemaker, brain stimulator). All participants will provide written informed consent before testing. Recruitment will be done via newspaper or Facebook advertisement. The study has been approved by the Ethics Committee of the Canton of Bern (Switzerland) and will be conducted according to the Declaration of Helsinki.

### Screening and group allocation

Participants will be screened over the telephone and only be invited to take part in the study if deemed eligible. Included participants will be assigned to one of two groups that will either receive an incentive during prospective memory tasks or not. Groups will be allocated using computer-generated random numbers, stratified by education, age, and sex.

### Study procedure

**Fig. 1:**
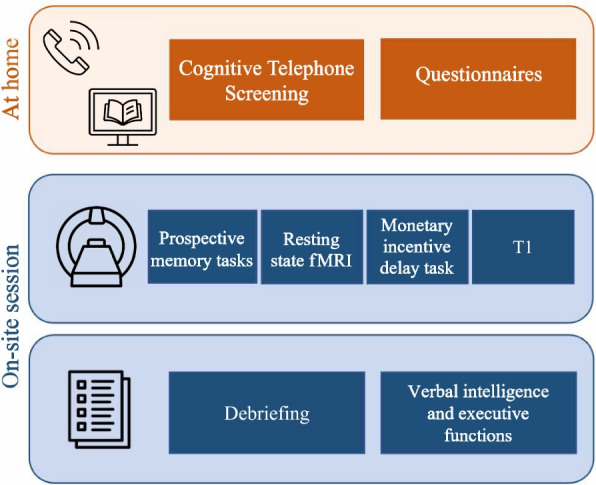
Study procedure

The study will consist of an online assessment of possible confounding factors using reliable and valid questionnaires as well as an on-site visit (Fig. 1). All participants will provide information on their monthly income, retirement status, monetary satisfaction and how often they donate money to charity. In addition, we will use the Edinburgh Handedness Inventory (EHI; [22]) to assess handedness and the NEO- Five Factor Inventory (NEO-FFI; [23]) to assess personality traits. To assess general sensitivity to reward or punishment, we will use the Behavioural Inhibition System/Behavioural Activation System Scales (BIS/BAS; [24]). Finally, to assess everyday prospective memory abilities, we will use the prospective and retrospective memory questionnaire (PMRQ; [25]) and the metacognitive prospective memory inventory (MPMI; [26]).

On the study date, we will acquire structural and functional MRI data using a 7-Tesla ultra-high-field Magnetom Terra scanner (Siemens Medical Systems, Erlangen, Germany). During fMRI, time-based and event-based prospective memory will be tested, followed by a modified version of the monetary incentive delay task (MID; [27]), which will be used to localize areas involved in incentive-related processing in general. In between the two fMRI tasks, resting state fMRI data will be acquired to allow participants to have a break before the next task. For task-based and resting-state fMRI, we will use a gradient echo sequence (TR = 1 s, TE = 24 ms, voxel size = 1.4 × 1.4 × 1.4 mm). T1-weighted anatomical images will be obtained using a MP2RAGE sequence (TR = 6 s, TE = 2.06 ms, voxel size 0.63 × 0.63 × 0.63 mm). We will acquire physiological data (i.e., heart rate and breathing) to control for noise [28].

After MRI data acquisition, we will ask participants to describe the prospective memory task in their own words to assess whether they remember what they had to do during the prospective memory tasks. Then they will rate the perceived importance, motivation, and difficulty of the tasks. Finally, they will complete a German vocabulary test to test their verbal intelligence [29] as well as the Trail Making Test [30] and the Digit-Symbol Substitution Test [31] to control for a possible influence of problems with executive functions.

### Prospective memory task

**Fig. 2:**
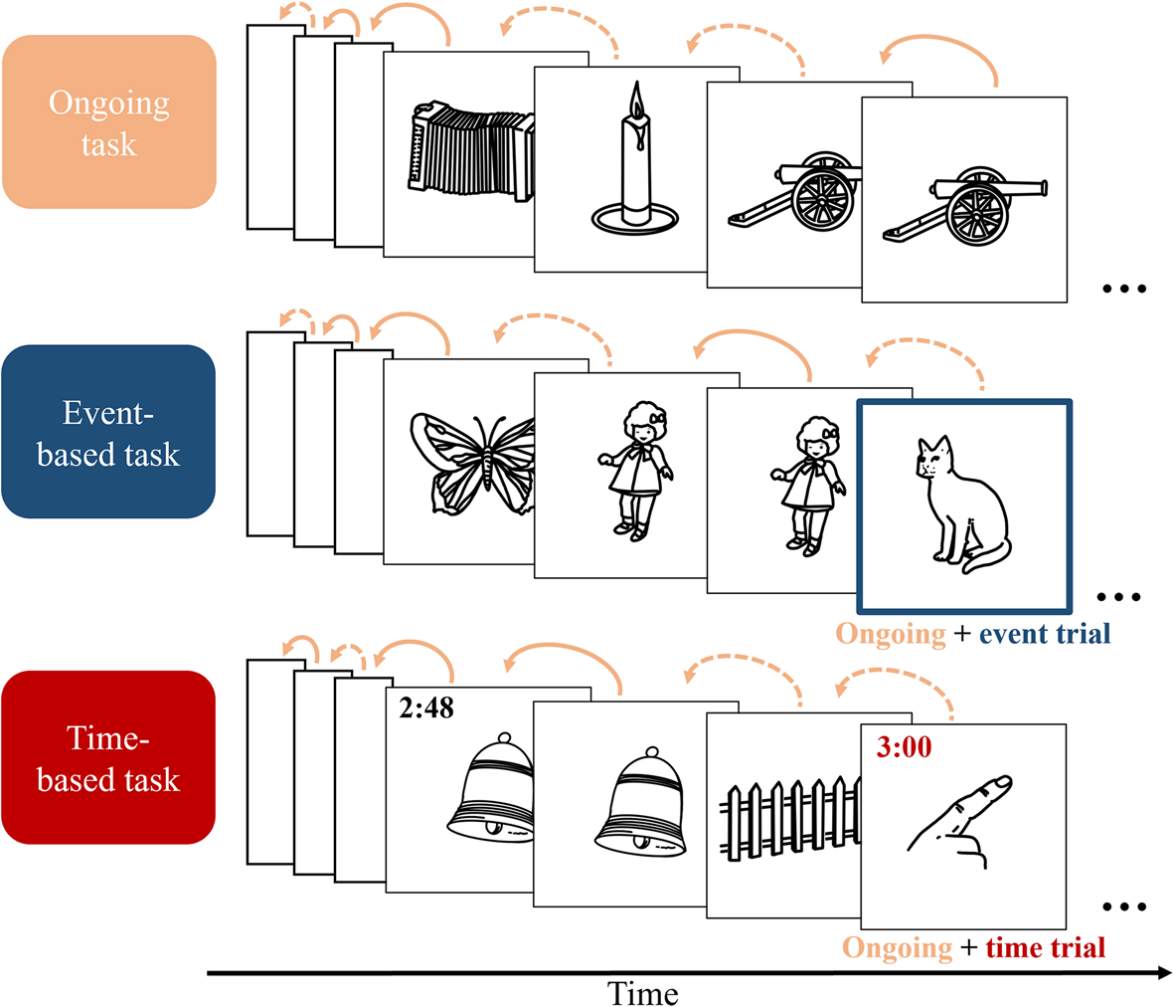
Procedure of the prospective memory paradigms. Each prospective memory task (i.e., event-based, or time-based) consists of an ongoing task (a 1-back task) either alone or in combination with a prospective memory task, where participants additionally need to respond to certain events (here: animals) or after a certain amount of time has passed (here: every minute). In the incentive group, participants will start with an initial amount of money (i.e., 5 Swiss Francs per task) and they will lose 0.50 Swiss Francs for every missed event or for every missed time-point

To examine the neural substrates of event-based and time-based prospective memory, we will use a computerized task in the MR scanner [32], which will be presented with PsychoPy (v2021.1.2). The task will consist of four blocks: During the first and the third block, participants will perform a 1-back working memory task (as an ongoing-task), during which they will need to decide whether the current image is the same as the image presented just before (Fig. 2). Pseudorandom sequences of 238 Snodgrass & Vanderwart [33] pictures will be displayed on a screen for 2–3 s, followed by a 1–2 s inter-stimulus interval, used as a temporal jitter. During the second and the fourth block, a prospective memory task will be added to the ongoing task. One block will contain the event-based task and the other block the time-based task. The order will be counterbalanced across participants. In the event-based task, participants are asked to press a button whenever an animal appears on the screen (Fig. 2). Every answer that occurs within 5 s following the presentation of an animal will be considered as a prospective memory hit [32, 34]. In the time-based task, participants are asked to press a button whenever one minute has passed (Fig. 2). To monitor the time, they will be able to press another key to display a clock for 3 s in the upper left corner of the screen. Every correct response occurring ± 2.5 s around each target time will be counted as a prospective memory hit [32, 34]. Each prospective memory block will consist of 158 trials; out of these, 54 trials will be ongoing 1-back hits (i.e., trials in which the picture will be identical to the previous one), and 10 prospective memory cues (i.e., one every minute at 1:00, 2:00, 3:00… until 10:00) so that both tasks are similar. Each prospective memory block will last 10.5 min.

To assess incentive-related processing during prospective memory tasks, participants in the incentive group will be informed that they are initially endowed with 5 Swiss Francs (per prospective memory task) that they may lose proportionally to the number of prospective memory cues they miss (0.50 Swiss Francs per missed cue). They will also be informed that half of their earnings will be donated to an organization of their choice (Doctors without borders, UNICEF, or WWF).

### Monetary incentive delay task

To examine the neural substrates of incentive-related processing in the absence of prospective memory, participants will complete a modified version of the monetary incentive delay task [27, 35] that consists of a baseline and an incentive block.

**Fig. 3:**
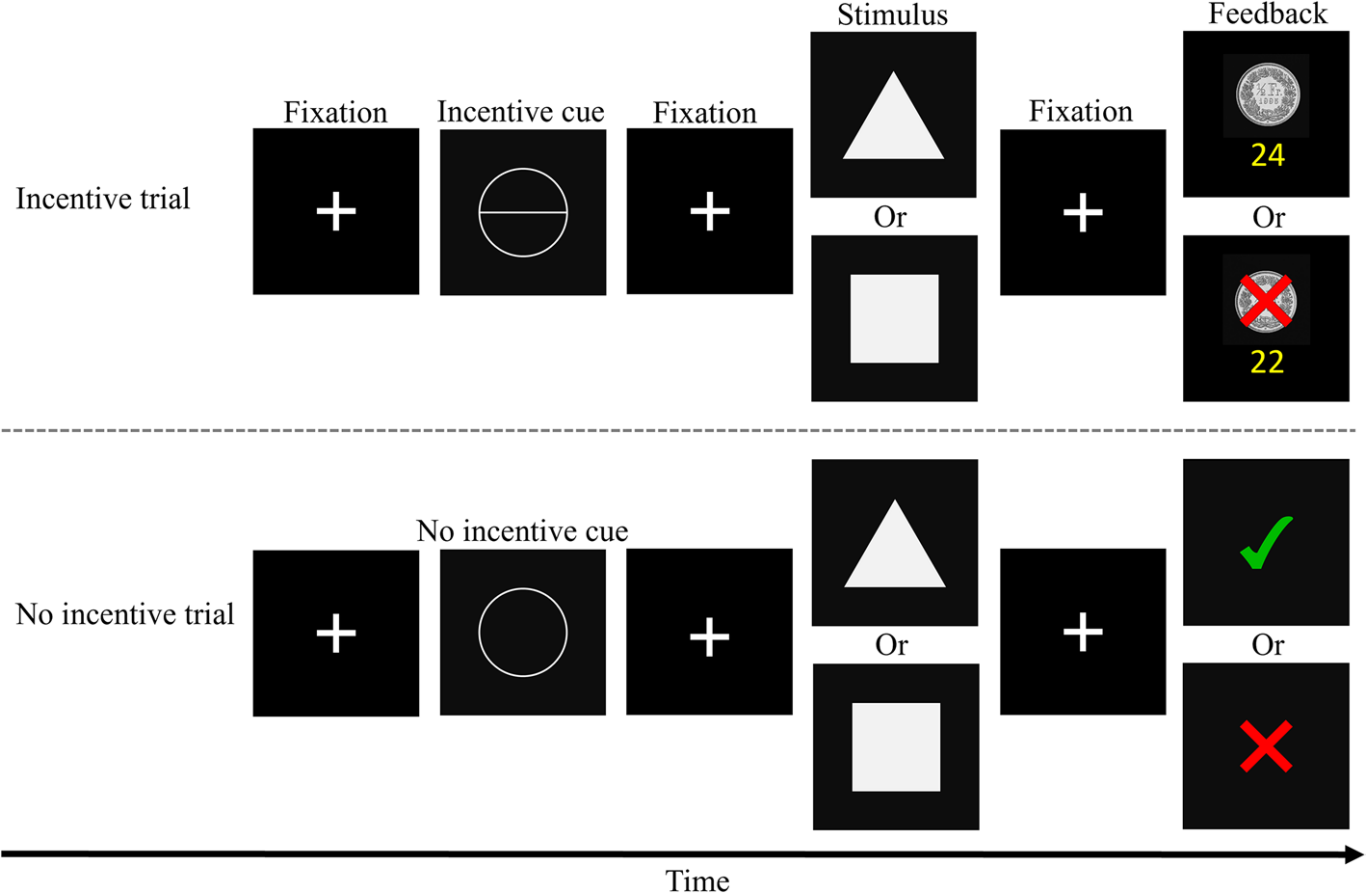
Procedure of the Monetary Incentive Delay task (baseline block not shown). The incentive block will consist of incentive trials and control trials. In each trial, participants will need to respond as fast and as correctly as possible to triangles or squares. During incentive trials (indicated by a circle with a horizontal line), they will be able to keep or lose points that will later be transferred to financial earnings. If they respond correctly and faster than during the baseline block, they will not lose a point (indicated by a centime coin). If they respond incorrectly or not fast enough, they will lose a point (indicated by a centime coin with a red cross). During control trials, it will not be possible to receive points, but participants will still receive feedback whether they responded correctly

At baseline, the participants will need to respond as fast and correctly as possible to triangles or squares and they will receive feedback (i.e., green tick = correct, red cross = incorrect or too late). Each trial will start with a fixation cross presented in the centre of the screen for 0.6—1.0 s. Then, either of the two stimuli will appear for 0.8 s, followed by another fixation cross (for 1.0—1.4 s) and feedback (for 0.5 s). After 20 trials, the mean response time of all correct answers will be calculated. Next, the incentive block will follow, during which the participants will again respond to triangles or squares (Fig. 3). In contrast to the baseline condition, however, they will be told that for some trials, it would now be possible to keep or lose an incentive. Each trial will start with the presentation of a fixation cross in the centre of the screen for 1—1.5 s, followed by a magnitude incentive cue for 2 s, and another fixation cross for 2—2.5 s. Then, participants will have to respond again to triangles or squares and, after another fixation cross, they will again receive feedback. The magnitude incentive cue will either be an empty white circle indicating a control trial, or a white circle with one horizontal line indicating an incentive trial. The two magnitude incentive cues will be presented 40 times each in randomized order. Hence, participants will complete a total of 80 trials. In order not to lose money, participants will need to answer correctly and faster than their mean response time during baseline. Control trials will be similar to the baseline condition. For incentive trials, answers that were correct and faster as during baseline will be indicated by a centime coin. A lost point (i.e., reaction time was slower than during baseline or an incorrect answer was given) will be indicated by a centime coin with a red cross. Below any 50-cent coin, the cumulative points will be shown. Participants will initially be endowed with an amount of 20 Swiss francs (40 points, each point corresponds to 0.50 Swiss Francs) and will be informed that they may lose it, depending on their response. We will encourage participants to respond as quickly as possible regardless of the type of the incentive cue. Comparable to previous studies, participants will have to keep more than 60% of total points (i.e., 24 points) to finally earn the incentive [27]. Otherwise, they will lose all the money. Again, half of their earnings will be donated to an organization of their choice.

### Statistical analysis

#### Behavioural: Do loss-related incentives enhance prospective memory?

Since we are primarily interested in whether incentives will enhance prospective memory, we will report accuracy and mean response times for the event-based task as well as accuracy and clock checking for the time-based task as primary outcomes.

First, we will test whether incentives will improve task accuracy. We will use two-way mixed-design ANOVA on correctly remembered trials, with the within-subject factor ‘task type’ (event-based or time-based) and the between-subject factor ‘group’ (incentive or control). Next, we will test whether response times in the event-based task will become quicker with an incentive. We will use one-way ANOVA on response times for correctly remembered trials, with the between-subject factor ‘group’ (incentive or control). For time-based prospective memory, we will test whether an incentive influences how often participants check the clock. Similar to our previous study [36], we will divide the 30 s before and after each target time into four intervals: T – 30 refers to the interval 30 to 15 s and T – 15 to the interval 15 to 0 s before target time. T + 15 or T + 30 then correspond to the intervals 0 to 15 s and 15 to 30 s after the target time. We will use a two-way ANOVA with the within-subject factor ‘time-interval’ and the between-subject factor ‘group’ (incentive or control). Finally, we will test whether monthly income, retirement status, personality traits, reward sensitivity, executive functions, or daily life prospective memory will predict performance. We will use linear regression with prospective memory accuracy as dependent variable and scores from the questionnaires as predictors. Age and gender will be included as covariates in all statistical analyses.

We will use R (Version 4.2.1) with Rstudio (Version 2022.02.3) for statistical analyses, with *p* < 0.05 considered statistically significant. We will correct for multiple comparisons using Tukey’s method. Whenever the assumptions of normality or homogeneity are not met, data will be transformed and/or non-parametric alternatives will be used. In case of missing data, we will use linear-mixed effects models rather than ANOVAs.

### Neuroimaging analysis

Task-based fMRI data will be pre-processed using Statistical Parametric Mapping (SPM12; Wellcome Trust Centre for Neuroimaging, London, UK), implemented in Matlab R2019b (MathWorks, Natick, MA, USA). Pre-processing will include realignment, slice-time correction, co-registration to the skull-stripped structural image, normalisation, and smoothing with a 6 mm FWHM Gaussian kernel. To remove low frequency noise from pre-processed data, a high-pass filter will be applied using SPM12’s default settings. For all first-level analyses, data will be analysed using a general linear model that models the time-series as a sequence of events convolved with the canonical hemodynamic response function provided by SPM12. We will analyse physiological data using either the CompCor [37] or RETROICOR approach, implemented in the TAPAS toolbox [38]. We will use the Computational Anatomy Toolbox (CAT12.8, [39]) for segmentation of T1 images.

### Are different brain regions involved in event-based vs. time-based prospective memory?

We will first analyse which brain regions are involved in event-based vs. time-based prospective memory. For the first-level analysis, we will fit one model for each prospective memory task. Each model will include three regressors, the first for ongoing trials during the ongoing task only block, the second for ongoing trials during the prospective memory block, and the third for prospective memory trials. We will build regressors using the onset of every correct answer. Task instructions will be modelled as regressors of no interest. We will define different contrasts corresponding to different prospective memory phases: 1) to obtain brain activity that is associated with maintenance of an intention, we will create contrasts between ongoing trials during the prospective memory block vs. ongoing only trials, 2) to obtain brain activity corresponding to the detection of a target, retrieval of an intention and its’ execution, we will create contrasts between prospective memory trials vs. ongoing trials during the prospective memory block. For time-monitoring, we will fit a model with the number of clock checks during the different time intervals previously described in the behavioural analysis. The onset will be defined as the moment in which the participant presses the key to check the clock. For time-monitoring, we will define contrasts between the different time intervals such as: T-15 vs. T-30, T-15 vs. T + 15, T-15 vs. T + 30, T-30 vs. T + 15, T-30 vs. T + 30, T + 15 vs. T + 30. Again, we will include six nuisance regressors for the six movement parameters as well as regressors of physiological data.

For second-level analyses, we will first use paired t-tests on first-level contrasts in the control group to test whether there would be differences in brain activity when comparing event-based to time-based prospective memory. Then, conjunction analysis will be performed to discern the neural substrates that are common in both task types. Then, we will calculate brain-behaviour correlations using beta estimates from significant regions correlated with task performance.

### Which brain regions are involved in incentive-related processing in general?

Next, we will test which brain regions are associated with incentives in general (i.e., without prospective memory). For first-level analysis, we will define a contrast between incentive and control trials during the anticipation phase of the monetary incentive delay task (i.e., the delay between cue appearance and target appearance). We will include six nuisance regressors for the six movement parameters as well as regressors of physiological data. For second level analysis, we will perform a one-sample t-test on first-level contrast (incentive vs. control) to find out which brain regions are associated with the anticipation of an incentive. Regions that will be identified with this analysis will be used as regions-of-interest (ROI) in subsequent second-level analyses of prospective memory.

### Which brain regions are involved in processing of incentives during prospective memory tasks?

Finally, we will test whether incentive-related processing during prospective memory tasks will be different to incentive-related processing during the monetary incentive delay task. We will first conduct two-sample t-tests on first-level contrast from the first analysis to test whether there are differences in brain activity with an incentive vs. without an incentive for event-based or time-based prospective memory. Next, we will use inclusive masks derived from the ROI analysis using the clusters identified in the monetary incentive delay task. Finally, we will use exclusive masking to test whether additional regions are active during incentive-related processing in prospective memory.

For all neuroimaging analyses, we will set the intensity threshold to *p* < 0.001 uncorrected, and the minimal cluster size threshold *k* to 15 voxels. For small regions (e.g., the hippocampus), we will mask voxels inclusively using generated masks and will set the intensity threshold to *p* < 0.001, uncorrected, with a cluster-size threshold *k* of 3 voxels. This is to reduce Type II errors associated with weak fMRI signal changes in small brain areas.

### Sample size calculation

For the determination of sample size, we used G*power [40]. We based calculations on a previous study that used incentives to enhance event-based prospective memory [12]. The effect size in this study (Cohen’s *f* = 0.25) suggests that an inclusion of *n* = 54 participants would be needed to find such effects in a repeated measures design with two groups and five measurements with *α* = 0.01 and a power of 1-β = 0.99. To account for attrition, drop-out or exclusion due to movement artefacts, we plan to recruit *N* = 60 participants (*n* = 30 in each group).

### Data management

We will pseudonymise all study data (i.e., participants will be given a unique participant number). The coding key will be stored separately and locked away. Each participant will be informed orally and in writing about the nature, usage, and storage of their data. Behavioural data will be stored in Dropbox folders encrypted with Boxcryptor. Neuroimaging data will be stored on GitLab hosted by servers of Bern University. Paper pencil data will be stored in folders that are locked away. Data processing will be done on personal computers/laptops and institutional servers. All computers will be password-protected and encrypted. The study team will be responsible for data management; data monitoring will be done by an independent researcher not involved in the study. At the end of the study, all personal data will be deleted. The procedures comply with Swiss data privacy laws.

## Discussion

The results of our study will provide insight into three important aspects of prospective memory in older adults. First, our study will reveal whether time-based and event-based prospective memory differ neuroanatomically. Second, we will find out whether we can enhance prospective memory by using an incentive and third, we will determine how incentives are processed in the brain during such tasks.

### What differentiates time-based prospective memory from event-based prospective memory?

Older adults typically perform better in prospective memory tasks in which monitoring demands are low [1, 32, 34, 41]. Time-based prospective memory tasks usually require more monitoring since there is no external reminder of what needs to be done and when. We use a hidden clock in our study and, therefore, our participants need to initiate clock checking by themselves. If participants perform worse in the time-based task compared to the event-based task, we will be able to determine at what point in the task their performance will become affected. It may be that they are well able to monitor the time by checking the clock, but they do not remember the actual intention (i.e., pressing a key when a minute has passed). This would indicate that an execution of many tasks at a time is too demanding for older adults and so they prioritise. Alternatively, they check the clock rather seldomly and therefore, their intentions are not accomplished very timely. This would support previous studies showing that older adults do not check the clock as efficiently as younger adults do and therefore, their time-based prospective memory is less accurate [36, 42]. In addition to dissecting at what point in the task participants’ performance drops, our data will examine the neuroanatomy underpinning time-based and event-based prospective memory. Any differences in brain activity between the two tasks would point to them being related but still biologically distinguishable. A previous study had reported that network activity was similar in time-based and event-based prospective memory [4]. However, it is worth noting that, in that study, the clock in the time-based task was always in plain view while in our study it will need a button press to appear. If hidden, participants may need to manage time similar to real life while a clock always in plain view may rather serve as an event monitor. Thus, we posit that our data will show different brain regions to be involved in either task. If, however, we do not find differences in brain activity between the two tasks, this may indicate that clock visibility in the time-based task does not play a role and that time-based and event-based tasks may activate similar brain areas regardless of the design of the time-based task. In any case, the results of network activity may later be transferred to non-invasive brain stimulation protocols to enhance or inhibit certain brain regions to enhance prospective memory.

### Do incentives modulate prospective memory?

Comparable to previous studies, we may find that incentives modulate event-based prospective memory. It has been shown, for example, that avoiding financial losses improved event-based prospective memory accuracy in older adults when it included a prosocial component (i.e., donation) [12]. We will extent this by showing whether time-based prospective memory accuracy can also be enhanced. If so, one could think of ways to implement this in real life. One example could be a bonus on insurance premium given to individuals by their health insurance companies that reflects behaviour, for example how they comply with medication or attend their doctor’s appointments. The value of the bonus would be reduced with each forgotten medical appointment, as was done in a previous study [43]. If we do not find an improvement by an incentive, this can either indicate that adding a prosocial component to an incentive does not enhance older adults’ motivation or that avoiding financial losses is not as motivating as hypothesized. Alternatively, task performance may be so good or so impaired that even if the participants are motivated by the incentive, they cannot improve any further. If performance without an incentive were similar (or better) than with it and the participants were not motivated by the incentive, this may indicate that intrinsic motivation was already very high. One reason could be that the participants wanted to prove to themselves that they were still cognitively fit. This would support the idea that intrinsic motivation plays an important role in healthy ageing [44].

### How are incentives processed in the brain during prospective memory tasks?

Finally, our study will provide data on how incentives are processed in the brain during prospective memory tasks. So far, the monetary incentive delay task has been mainly used to study incentive-related processes in the brain. This task differentiates anticipation of an incentive from the actual outcome (i.e., a phase in which one receives feedback about an incentive). This differentiation is not possible in a prospective memory task because any feedback would serve as a prospective memory cue and would therefore bias actual prospective memory abilities. It is, however, possible to assess the anticipation of an incentive since participants are informed in the beginning of the task that they may receive an incentive depending on their performance. During anticipation in the monetary incentive delay task, older adults showed less activity in the ventral and anterior insula as well as the dorsal striatum than younger adults did [16, 45–49]. It is not fully understood how specific any activity reduction in the ventral and anterior insula is for gains or losses, or the monetary incentive delay task in general. Some studies found lower activity in these areas for both gains and losses [16, 45–49]. Others found that lower activity in the anterior insula was specific to the avoidance of losses. And still others did not find any activity differences between gains and losses at all [18]. So it could be that the brain responds less in general when older adults anticipate an incentive. It is not well known whether activity reductions are specific to the monetary incentive delay task or whether they can be generalised to other types of tasks. Since we will use both the monetary incentive delay task and two different prospective memory tasks, our study will provide new insights into how incentives are processed in the brain in older adults when different tasks are used.

### Limitations

Our study may have some limitations. One limitation might be that the small amount of money that participants can earn might not be motivating enough. At least for donation, we highlighted the difference even a few Swiss Francs will make by showing pictures of aid organisations and examples of what they can do with small amounts of money. Another limitation might be that ventral and subcortical brain regions are in vicinity to bone and sinuses which makes them vulnerable to artefacts caused by magnetic field inhomogeneity, particularly with ultra-high field MRI [50]. We will use a small voxel size (in our study 1.4 mm) to account for that. Another limitation might be that the incentive anticipation during the prospective memory task is different to incentive anticipation in the monetary incentive delay task, as the incentive will only be anticipated in the beginning of the task and not repeatedly during the task. So it could be that we do not find activation in the prospective memory task in the ROIs that were defined in the monetary incentive delay task. Finally, the use of a more traditional time-based task has the advantages we already discussed, but one disadvantage could be enhanced movement since the participants have to use an additional finger to check the clock.

## Conclusion

Our study will test the neuroanatomical correlates of event-based and time-based prospective memory in older adults. We will, in addition, examine whether incentives can enhance event-based and time-based prospective memory and how it is processed during such tasks. The results of our study will be an important step towards a better understanding of how memory changes when we get older and for developing interventions to counteract cognitive decline.

## Study status

Recruitment of participants started in January 2022. Data acquisition is ongoing, and we expect to finish data acquisition in autumn 2023.

## Data Availability

The results of this study will be published in an open access journal. The datasets supporting the conclusions of the article will be made publicly available in an open access system that is hosted by the University of Bern after publication of the manuscript.

## Abbreviations

CAT: Computational Anatomy
fMRI: Functional Magnetic Resonance Tomography
MRI: Magnetic Resonance Tomography
COGTEL: Cognitive Telephone Screening
EHI: Edinburgh Handedness Inventory
NEO-FFI: NEO- Five Factor Inventory
BIS/BAS: Behavioural Inhibition System/Behavioural Activation System
PMRQ: Prospective and Retrospective Memory Questionnaire
MPMI: Metacognitive Prospective Memory Inventory
MID task: Monetary Incentive Delay task
MID: Monetary Incentive Delay
UNICEF: United Nations International Children’s Emergency Fund
WWF: World Wide Fund for Nature
ROI: Region of interest
